# Unraveling the Complexity of Amyotrophic Lateral Sclerosis Survival Prediction

**DOI:** 10.3389/fninf.2018.00036

**Published:** 2018-06-14

**Authors:** Stephen R. Pfohl, Renaid B. Kim, Grant S. Coan, Cassie S. Mitchell

**Affiliations:** ^1^Department of Biomedical Engineering, Georgia Institute of Technology, Emory University School of Medicine, Atlanta, GA, United States; ^2^Department of Biomedical Informatics, Stanford University, Stanford, CA, United States; ^3^Medical Scientist Training Program, University of Michigan Medical School, Ann Arbor, MI, United States; ^4^School of Medicine, University of Texas Health Science Center at San Antonio, San Antonio, TX, United States

**Keywords:** predictive medicine, data complexity, survival analysis, neuromuscular disease, health informatics

## Abstract

**Objective:** The heterogeneity of amyotrophic lateral sclerosis (ALS) survival duration, which varies from <1 year to >10 years, challenges clinical decisions and trials. Utilizing data from 801 deceased ALS patients, we: (1) assess the underlying complex relationships among common clinical ALS metrics; (2) identify which clinical ALS metrics are the “best” survival predictors and how their predictive ability changes as a function of disease progression.

**Methods:** Analyses included examination of relationships within the raw data as well as the construction of interactive survival regression and classification models (generalized linear model and random forests model). Dimensionality reduction and feature clustering enabled decomposition of clinical variable contributions. Thirty-eight metrics were utilized, including Medical Research Council (MRC) muscle scores; respiratory function, including forced vital capacity (FVC) and FVC % predicted, oxygen saturation, negative inspiratory force (NIF); the Revised ALS Functional Rating Scale (ALSFRS-R) and its activities of daily living (ADL) and respiratory sub-scores; body weight; onset type, onset age, gender, and height. Prognostic random forest models confirm the dominance of patient age-related parameters decline in classifying survival at thresholds of 30, 60, 90, and 180 days and 1, 2, 3, 4, and 5 years.

**Results:** Collective prognostic insight derived from the overall investigation includes: multi-dimensionality of ALSFRS-R scores suggests cautious usage for survival forecasting; upper and lower extremities independently degenerate and are autonomous from respiratory decline, with the latter associating with nearer-to-death classifications; height and weight-based metrics are auxiliary predictors for farther-from-death classifications; sex and onset site (limb, bulbar) are not independent survival predictors due to age co-correlation.

**Conclusion:** The dimensionality and fluctuating predictors of ALS survival must be considered when developing predictive models for clinical trial development or in-clinic usage. Additional independent metrics and possible revisions to current metrics, like the ALSFRS-R, are needed to capture the underlying complexity needed for population and personalized forecasting of survival.

## Introduction

The prolific 2014 ALS Association’s Ice Bucket Challenge commenced the world-wide dumping of ice water on the heads of courageous supporters to bring awareness and research funding to a lesser-known yet fatal neurodegenerative disease, Amyotrophic Lateral Sclerosis (ALS) (Wicks, 2014). Despite recent identification of genotypes (Hrastelj and Robertson, 2016) and phenotypes (Hollinger et al., 2016), ALS remains elusive, largely due to its heterogeneous nature. The multi-faceted etiology of ALS is reflected in its sporadic underpinnings, as less than 10% of patients have a familial form (Hrastelj and Robertson, 2016). Varied symptom onset types include extremity muscle deficits, the dominant “limb onset”; dysarthria or dysphagia symptoms, “bulbar onset”; and a small fraction have onset with respiratory or generalized weakness. Finally, post-onset ALS survival duration fluctuates from less than 1 year to over 10 years (Beghi et al., 2011).

Inherent heterogeneity of ALS makes it exceedingly difficult to predict and exacerbates anxiety among ALS patients and their families. Heterogeneity also increases the physicians’ challenge to develop a timely intervention plan and is blamed for numerous failed clinical trials, as statistical variance drowns all but inconceivable treatment effect sizes (Beghi et al., 2011). The inability to accurately gauge varied outcomes makes the assessment of therapeutic efficacy problematic, slowing curative discovery (Rutkove, 2015). In short, the ability to account for heterogeneity and predict real-time ALS clinical outcomes is a necessity.

Recent crowdsourcing efforts (Atassi et al., 2014; Küffner et al., 2015; Zach et al., 2015) focused on developing computer models to predict clinical ALS progression. Namely, the DREAM-Phil Bowen ALS Prediction Prize4Life challenge (Küffner et al., 2015) used the PRO-ACT clinical trial data (Atassi et al., 2014) to forecast slope of decline of the Revised ALS Functional Rating Scale (ALSFRS-R) (Cedarbaum and Stambler, 1997; Cedarbaum et al., 1999; Castrillo-Viguera et al., 2010; Creemers et al., 2015), a clinical questionnaire that evaluates externally observable function. However, the ambiguity and heterogeneity associated with ALSFRS-R (Voustianiouk et al., 2008; Franchignoni et al., 2013, 2015; Mandrioli et al., 2015) as an outcome metric and the characteristics of the PRO-ACT cohort (Fournier and Glass, 2015) limit their translational usage. A very recently published multi-clinic study was one of the first to develop a personalized patient prediction model (Westeneng et al., 2018) for patients who did not receive ventilation support. While the latter study was yet another step forward, patient heterogeneity still greatly complicates personalized prediction, much less population-level models necessary for clinical trials.

Before truly representative and personalized or population-level prediction models can be a new standard for clinical trial development and in-clinic decision-making, we must first better understand the complex relationships among the clinical metrics used to characterize ALS progression and forecast survival. ALS is a diagnosis of exclusion, meaning there is not one single objective medical “test” (imaging, blood test, genotyping, etc.) that is applicable to positively identifying all ALS patients nor is there one single “measurement” that can be used to objectively and precisely stage the greatly variable temporal ALS disease course. For this reason, a combination of assessments must be used to characterize clinical ALS. Understanding the relationships among existing clinical assessments and how they correlate to various disease stages is critical to optimize clinical ALS characterization, and ultimately, interventional and survival forecasting.

The primary goals of this study are to: (1) assess the underlying complex relationships among clinical ALS metrics; (2) identify the “best” ALS clinical predictors and how predictor ranking changes as a function of disease progression and/or disease stage. First, we utilize raw clinical ALS assessment metrics and patient characteristics to assess the complexity of the disease, including the underlying latent dimensionality. Secondly, we construct interactive models that directly utilize survival/lifespan as the outcome metric for ALS patient survival classification at various thresholds ranging from 30 days to 5 years. Our analytical findings provide key insight for developing better ALS progression metrics and resolving the heterogeneity of ALS that plagues clinical trials and blurs clinical decision-making.

## Materials and Methods

We performed a retrospective analysis of 8,028 clinical visit records collected from 1,585 patients at the Emory ALS Clinic (Emory University Hospital, Atlanta, GA, United States). Data collection, transcription, and quality control methods are as previously published (Mitchell et al., 2015a; Hollinger et al., 2016). All data was de-identified and anonymized. Institutional Review Board approval was obtained from Georgia Institute of Technology and Emory University.

**FIGURE 1:**
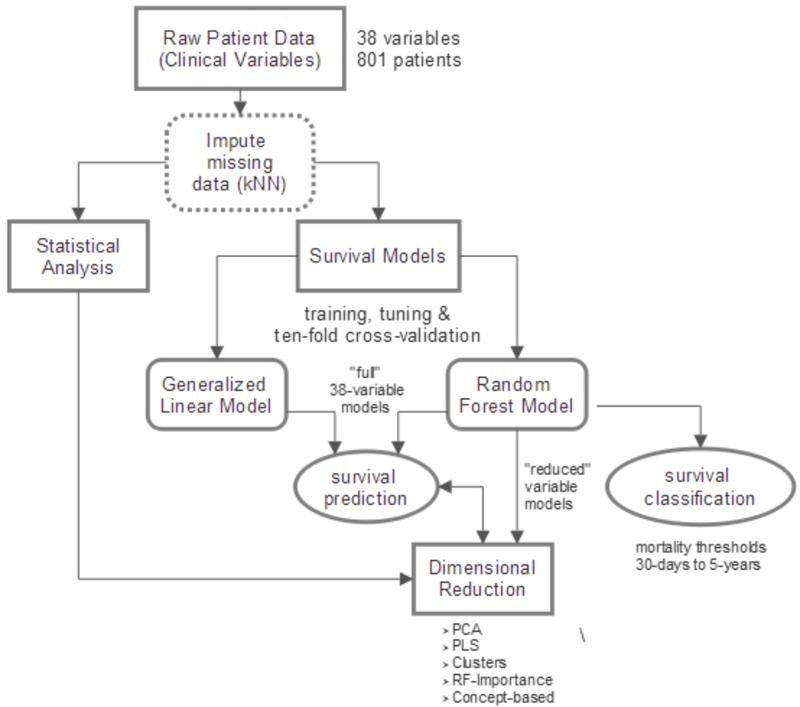
Overview of study design. Raw clinical data (801 patients, 38 variables) is imputed using *k*-nearest neighbors (kNN) technique. A combination of statistical analysis of the clinical data, constructed survival prediction and classification models, and dimensionality reduction is utilized to accomplish two main goals: (1) obtain a comprehensive view of complex clinical relationships; (2) determine which clinical variables are the “best” predictors of survival and, namely, how their predictive influence changes with disease progression.

We first performed an exploratory analysis of the raw clinical data. We then construct interactive models to predict and/or classify survival duration. Collectively, the analysis of raw data and the models are used to assess the complex relationships among clinical measures of ALS and their dynamic correlation with survival forecasting. **Figure 1** provides a diagrammatic overview of the study’s methods with additional details presented below.

### Data Inclusion Criteria and Missing Value Imputation

The 38 clinical variables utilized as predictors are listed in the left column of **Table 1**. Included patients had a known date of death and a complete set of Medical Research Council (MRC) muscular scores measured for at least one visit. The final data set (**Table 1**) for model development encompassed 3,918 visit records collected from 801 patients. Missing values in the original data set were imputed using the *k*-nearest neighbor (kNN) imputation technique. Specifically we utilized the mean value for the 5-nearest neighbors (e.g., kNN *k*-value of 5), with a Euclidean distance metric as described in Ishwaran et al. (2008).

**Table 1:** Number of missing visit records by field.

Variable	Missing records –	Missing records –
	all (%)	valid (%)
Bicep.Left	3238 (39.8)	1451 (37.0)
Bicep.Right	3237 (39.8)	1446 (36.9)
Delt.Left	3240 (39.9)	1456 (37.2)
Delt.Right	3236 (39.8)	1452 (37.1)
Edb.Left	4869 (59.9)	2374 (60.6)
Edb.Right	4851 (59.7)	2347 (59.9)
Gas.Left	3911 (48.1)	1905 (48.6)
Gas.Right	3896 (47.9)	1900 (48.5)
Ham.Left	3475 (42.7)	1639 (41.8)
Ham.Right	3480 (42.8)	1641 (41.9)
Intrin.Left	3580 (44.0)	1657 (42.3)
Intrin.Right	3573 (43.9)	1657 (42.3)
IP.Left	3414 (42.0)	1589 (40.6)
IP.Right	3419 (42.1)	1587 (40.5)
Quad.Left	3385 (41.6)	1569 (40.0)
Quad.Right	3383 (41.6)	1566 (40.0)
TA.Left	3464 (42.6)	1619 (41.3)
TA.Right	3449 (42.4)	1608 (41.0)
Tricep.Left	3299 (40.6)	1501 (38.3)
Tricep.Right	3290 (40.5)	1490 (38.0)
Wrist.Left	3458 (42.5)	1592 (40.6)
Wrist.Right	3445 (42.4)	1580 (40.3)
Time.Since.First.Visit	0 (0.0)	0 (0.0)
Time.Until.Death	3495 (43.0)	0 (0.0)
Duration.From.Diagnosis	467 (5.7)	59 (1.5)
Duration.From.Onset	105 (1.3)	39 (1.0)
Forced.Vital.Capacity	2718 (33.4)	1226 (31.3)
% Predict	2502 (30.8)	1108 (28.3)
NIF	3388 (41.7)	1459 (37.2)
O2.sat	5086 (62.6)	2345 (59.9)
Age	0 (0.0)	0 (0.0)
Patient.Weight	5205 (64.0)	2114 (54.0)
Patient.Info.Height	3931 (48.4)	1590 (40.6)
ALSFRS.r.total	5076 (62.4)	2611 (66.6)
ALSFRS.r.ADL	6615 (81.4)	2996 (76.5)
ALSFRS.r.Respiratory	6316 (77.7)	2818 (71.9)
Onset.Type	221 (2.7)	112 (2.9)
Sex	0 (0.0)	0 (0.0)

*The Valid designation refers to those records belonging to patients in which the time of death was known and for which muscular measurements were recorded for at least one visit*.

Metrics included are: forced vital capacity (FVC); the observed percent of predicted FVC (% predict FVC); the negative inspiratory force (NIF); oxygen saturation (O_2_ sat); left and right sided MRC muscular function scores for eleven extremity muscles; height; weight; onset type (bulbar or limb); sex; age; the (ALSFRS-R) (Cedarbaum and Stambler, 1997; Cedarbaum et al., 1999), including the ALSFRS-R total score, the ALSFRS-R activities of daily living (ADL) sub-score, and the ALSFRS-R respiratory sub-score; estimated date of ALS symptom onset (as reported by patient); ALS diagnosis date; ALS “baseline” visit date (first visit at tertiary ALS clinic); and date of death.

### Exploratory Clinical Data Statistical Analysis

The linear Pearson correlation was computed for each pair of visit-level variables for the 3,918 visit records and visualized as a correlation matrix using *corrplot* (Wei and Simko, 2016). To produce the dendrogram, correlations were re-calculated with time until death (the response variable) excluded. The resulting pairwise correlations were transformed to a distance metric such that distance was (1-correlation)/2 and clustered hierarchically with complete linkage.

The dimensionality of the imputed predictor dataset was reduced with principal component analysis (PCA) and results visualized with *FactoMineR* and *factoextra* (Lê et al., 2008). Additionally, a partial least square (PLS) model (Dayal and MacGregor, 1997; Friedman et al., 2010) was fit with visit-level variables with time until death as the response variable. The projection of a variable onto a PLS component was taken as the corresponding element of PLS weight matrix [matrix **R** in (Dayal and MacGregor, 1997)].

### Overview of Survival Prediction Models

Two primary predictive model types were constructed: a generalized linear model (GLM) where the regularization path is computed using the elastic net penalty (e.g., a linear combination between lasso and ridge regression) (Friedman et al., 2010), abbreviated in the figures as “Lasso,” and the full random forest model (Liaw and Wiener, 2002), depicted in the figures as “Full-RF. Both the GLM model and the Full-RF included all 38 variables illustrated in **Table 1**. The GLM and random forest model types were chosen based on their usage in prior ALS survival prediction literature (Fournier and Glass, 2015; Küffner et al., 2015), as well as their unique attributes that make them suitable to the goals of this study. The GLM enables *p*-values to be calculated to examine quantitative significance of clinical variables as predictors for survival. The main disadvantages of the GLM are that it does not account for complex interactions, and the GLM implicitly assumes the input variables fit a multivariate normal distribution. In contrast, the random forest models do account for complex interactions, do not require the assumption of a multivariate normal distribution, are non-parametric and robust under arbitrary re-scalings of the inputs (Svetnik et al., 2003). In summary, random forest models are simply better suited for examining temporal dynamics and for elucidating data structure complexity with model dimensionality reduction techniques (Strobl et al., 2008).

For the stated goals of this study, which focuses on examining complex clinical ALS metric relationships and their varying correlation with survival as a function of disease progression, using independent records was preferable to using patient-specific histories (e.g., patient-specific variable slopes) to minimize over-fitting. Therefore, all survival models predict time until death using independent visit records. Specifically, time until death (in days) for each visit was considered as the response variable with the visit-level variables (**Table 1**) as predictors. Both predictive regression and classification random forest models of survival were constructed. Using standard random forest classification models, mortality was predicted as a binary outcome across mortality thresholds (**Table 2**) in which a positive result is assigned to visits with a time until death smaller than the threshold time. Random forest out of bag predictions for the final model were used to estimate sensitivity (recall), specificity, precision (positive predictive value) across the spectrum of internal decision cut-offs and mortality thresholds.

**Table 2:** Number of visits and patients across mortality thresholds.

Mortality threshold	Number of visits (%)	Number of patients (%)
30 days	91 (2.3)	85 (10.6)
60 days	194 (5.0)	172 (21.5)
90 days	324 (8.3)	283 (35.3)
180 days	721 (18.4)	491 (61.3)
1 year	1506 (38.4)	639 (79.8)
2 years	2565 (65.5)	732 (91.4)
3 years	3101 (79.1)	766 (95.6)
4 years	3396 (86.7)	782 (97.6)
5 years	3591 (91.7)	789 (98.5)

*Data shown are the number of visits in the dataset with a time until death of less than associated mortality threshold and the patients associated with those visits*.

#### Reduced Models Using Dimensionality Reduction

In machine learning, dimensionality reduction is the process of reducing the number of model variables under consideration by obtaining a set of principal variables from the full set of variables that can be used to accurately predict outcome. Dimensional reduction is typically divided into feature selection and feature extraction. In short, the primary purpose of dimensional reduction in the present study is to better elucidate underlying characteristics of the data set and what variables most contribute to model performance (e.g., better survival prediction).

We used four standard dimensionality reduction settings to iteratively examine predictive performance in reduced random forest models with *k* components, “PCA-RF” utilized projections of data on to the principal components as predictors in the random forest (Tolosi and Lengauer, 2011); “PLS-RF” used the PLS scores (Dayal and MacGregor, 1997) as predictors in the random forest; “Importance” used rank-ordering of variable importance values from the Full-RF to reduce the number of predictors (Strobl et al., 2007); “Clusters” used the clustered features from *supervised feature clustering* (Tolosi and Lengauer, 2011) as predictors to random forest. No explicit specifications are made regarding the number of dimensions included in the reduced models. Instead, the relative performance of the models for a grid of potential reduced model component values, *k*, is performed and illustrated for all four dimensionality reduction methods. This allows visualization of the necessary dimensionality to attain performance comparable to that of the full random forest model, “Full-RF,” which uses all 38 clinical variables.

In addition to reduced models constructed using standard dimensionality reduction techniques as described above, a reduced model variable sub-set combination experiment was performed using concept-based variables grouped by clinical concept: “duration” (time from onset, diagnosis, and first visit); “age”; “respiratory” (O_2_ saturation, NIF, FVC, and % predicted FVC); “muscle” (all individual muscle scores), “ALSFRS” (ALSFRS-R total, respiratory and ADL sub-scores); “weight and height”; “onset type and sex” (bulbar or limb; male or female). Concept-based variable grouping assists in understanding the importance of variable sub-sets that are clinically intuitive.

#### Survival Model Construction and Validation

All regression and classification models were built with the *caret* framework in the R programming language. Random forest models were built with the *randomForest* (Liaw and Wiener, 2002) package and generalized linear models were built with the *glmnet* package (Friedman et al., 2010). For each model, 10-fold cross validation was repeated ten times. The mean and standard deviation for model performance metrics are computed based on pooling the performance results across the ten repetitions of 10-fold cross validation, corresponding to 100 values.

For ten repetitions of 10-fold cross-validation, the original sample is first randomly partitioned into ten equal size subsamples. Of the ten subsamples, a single subsample is retained as the validation data for testing the model, and the remaining subsamples are used as training data. The cross-validation process is then repeated ten times (the folds), with each of the subsamples used exactly once for validation. The results from the folds are then combined to produce a mean and standard deviation for each performance metric. The advantage of this method is that it maximizes the analytical sample size as all observations are used for both training and validation, and each observation is used for validation exactly once. For this exploratory study to assess clinical ALS dimensionality and complexity, 10 repetitions of 10-fold cross-validation was deemed superior to a truly split training and validation data, the latter which is better for non-exploratory models solely focused on personalized prediction accuracy.

In regression cases, performance is defined as either the root mean squared error (RMSE) or the testing *R*^2^ (squared correlation or percent variance explained) of the predicted response for testing samples with respect to their known value. In classification cases, the performance was defined as the area under the curve (AUC) of the receiver-operating-characteristic (ROC) for testing samples.

For each testing-training split of the cross-validation procedure, the missing data were imputed using kNN technique and scaled to zero-mean and unit-variance. This process was repeated over a grid search of the tuning parameters (α and λ for elastic net, m_try_ for random forest) to determine the parameters that result in optimal performance (minimal RMSE or maximal AUC). Other relevant random forest parameters followed the defaults from the *randomForest* R package (Liaw and Wiener, 2002); that is, 500 trees were used for each forest and trees were grown to maximal depth subject to the constraints that terminal nodes had a minimum of size 5 for regression tasks and of size 1 for classification tasks. In each case, a final model was then generated with the optimal parameters for the full dataset after missing value imputation using kNN. If unspecified, data are presented as mean ± standard deviation and the results visualized with *ggplot* (Wickham, 2009).

#### Assessment of Survival Model Variable Importance

The importance of variables in the random forest models was assessed as the percent increase in the out-of-bag mean squared error upon independent permutation of each variable for the final model following cross validation with the supervised feature clustering method used to correct for potential correlation bias (Tolosi and Lengauer, 2011). The significance of variables in the GLM model was assessed with the exact post-selection inference method for the lasso (Tibshirani et al., 2014), enabling *p*-value computation while controlling for implicit multiple comparison.

## Results

A total of 801 deceased patients, 3,918 clinic visits, and 38 assessed clinical variables (**Table 1**) were ultimately included in the results and analysis of this study. As depicted in **Figure 1**, this exploratory study consists of analysis of complex relationships within the raw data, development of survival prediction and classification models, and dimensionality reduction of the raw data and survival models. The resulting overlapping insights bring into focus a comprehensive view of the complex relationships among variables currently utilized to assess disease progression and predict ALS patient survival.

### Exploratory Clinical Data Analysis

We begin with an exploratory analysis of the raw clinical data to examine relationships, correlations, complexity, and variance that can impart clinical and theoretical insight.

#### Correlation of Visit-Level Variables (Clinical Metrics)

**FIGURE 2:**
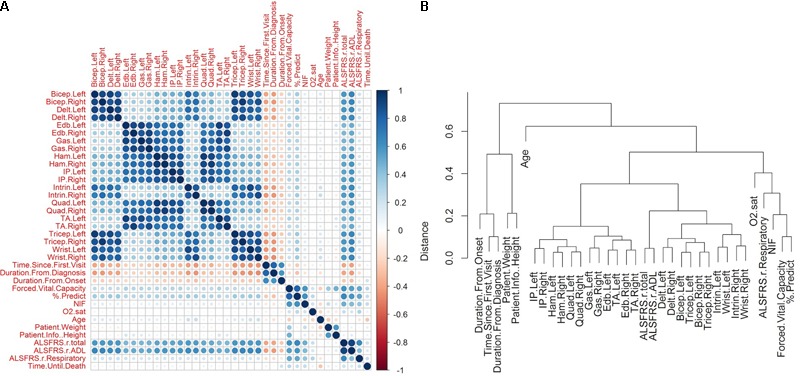
Correlations between raw amyotrophic lateral sclerosis (ALS) clinical variables. The raw clinical data was filtered to include only those patients in which the time of death was known and for which muscular measurements were recorded for at least one visit (*N* = 801, variables = 38). Missing values were imputed using kNN technique (see section “Materials and Methods”). **(A)** The pairwise Pearson correlation was computed for each pair of variables following kNN imputation. The matrix illustrates the sign and magnitude of correlation for each pair. **(B)** The correlations were transformed to a distance metric (see section “Materials and Methods”) and clustered hierarchically with complete linkage. Time of death was excluded from the clustering so as to obtain a representation of the correlation structure of the predictor data.

The visualization of the correlation structure of the raw data reveals several high level relationships among the visit-level variables (**Figures 2A,B**). In particular, there is a high degree of correlated decline (*r* ≈ 0.7–0.9) in the upper body muscles (bicep, tricep, deltoid, wrist, intrinsic), which occurs largely independently of an analogously correlated decline of the lower body muscles (hamstring, quadriceps, gastrocnemius, iliopsoas, extensor digitorum brevis, transverse abdominis), as is evident by the lesser correlation (*r* ≈ 0.3–0.45) between the upper and lower scores.

The respiratory measures of FVC, % predicted of FVC, and NIF tend to correlate together (*r* ≈ 0.6–0.8). The FVC and the % predicted of FVC show mild correlations with the muscle scores (*r* ≈ 0.25–0.35), while the relationship between NIF and the muscle scores is weaker (*r* ≈ 0.07–0.15). The oxygen saturation shows a mild relationship with FVC, % predicted of FVC, and NIF (*r* ≈ 0.20–0.25), but has very little relationship with muscular scores (*r* ≈ 0–0.06).

The ADL sub-score of ALSFRS-R moderately correlates with the muscular measures (*r* ≈ 0.5–0.65), while the respiratory sub-scores show a weak relationship with the muscle scores (*r* ≈ 0.16–0.22). Interestingly, the ADL and respiratory ALSFRS-R sub-scores correlate comparably with FVC, % predicted FVC, and NIF (ALSFRS-ADL: *r* = 0.548, 0.587, 0.432; ALSFRS-Respiratory: *r* = 0.504, 0.519, 0.386), with a slightly larger degree of correlation between the respiratory measures and ADL sub-score than with the respiratory measures and ALSFRS-R respiratory sub-score.

Negative correlations with the *disease start variables* (time since first visit, duration from diagnosis, duration from onset) implies that the variable in question declines after the start of disease. For each declining variable, the duration from onset shows a lesser magnitude of negative correlation than time since first visit (e.g., baseline). There is lesser correspondence between the visit-level variables and the time until death. The muscle scores, which showed a moderate degree of decline with respect to the disease start variables, have very little correlation with the time until death (*r* ≈-0.03–0.09). In contrast, the respiratory measures, which show a lesser degree of decline with respect to the disease start, have a somewhat stronger correlation with the time until death (FVC: *r* = 0.28; % predicted FVC: *r* = 0.22; NIF: *r* = 0.24). There are mild correlations of the time until death with ALSFRS (total: *r* = 0.21; ADL: *r* = 0.15; respiratory: *r* = 0.23) and with age (*r* = -0.27). The lack of any large correspondence of any one variable with the time until death implies that a complex interaction of measures may be required for accurate survival prediction.

#### Exploratory Dimensionality Reduction of Raw Clinical Variables

**FIGURE 3:**
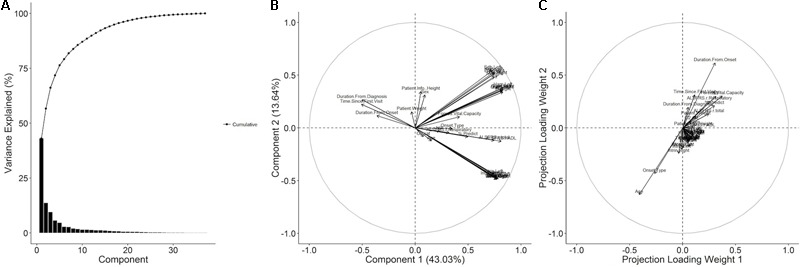
Principal component and partial least squares (PLS) decomposition of raw ALS clinical variables. **(A)** The raw ALS clinical dataset was imputed and then decomposed with principal component analysis (PCA) and the % variance explained by each component plotted. The variables were projected onto **(B)** the first and second principal components. A PLS model with the time until death as the response was generated and the columns of the weight matrix visualized as variable projections for **(C)** the first and second columns.

Decomposition of the visit-level predictor variables (all visit-level variables with the exception of time until death) with PCA gives an alternate representation of the data in terms of a reduced number of orthogonal components. In this representation, the first and second principal components account for 43.03 and 13.64% of the variance with 91.06% of the variance accounted for by the first thirteen components (**Figure 3A**). The projections of the variables onto the first two components (**Figure 3B**) reveal that the majority of the variance in the predictor data is due to variance in the muscle scores and the ALSFRS-R total and its ADL sub-score. The third and fourth components (Supplementary Figure S1A) account for variance in the respiratory visit data and patient characteristics. An analogous partial-least decomposition of the variables was generated through a PLS model for time until death as the response variable. In this case, age, onset type, and the duration from onset have the most prominent contributions to the first four components (**Figure 3C** and Supplementary Figure S1B), implying that those variables may account for much of the predictive signal in the context of the PLS model.

### Survival Models to Explore Clinical Variables as Predictors

**FIGURE 4:**
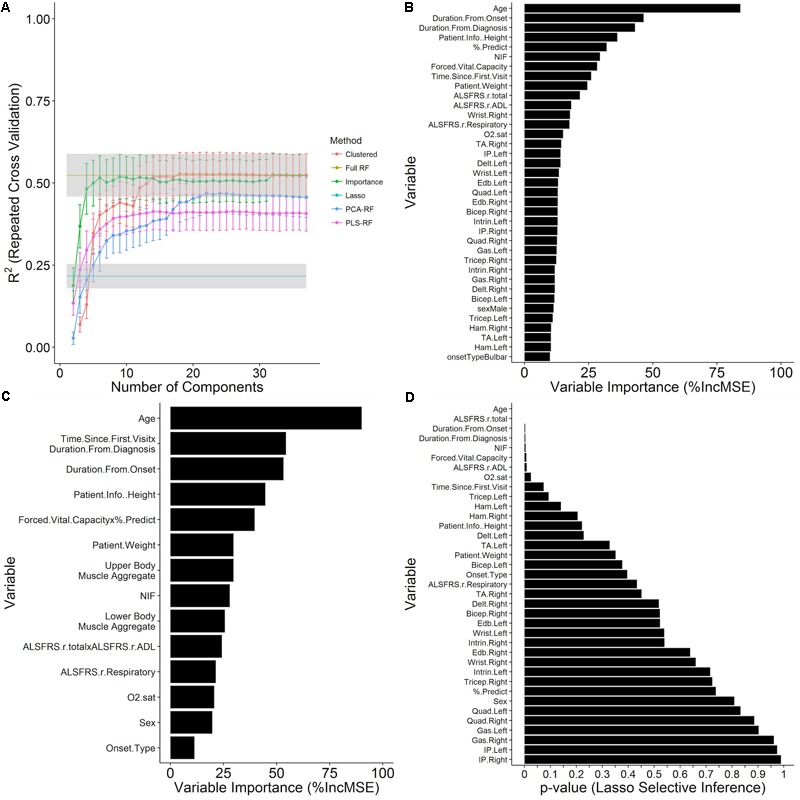
Prediction of time until death using survival models constructed from independent visit records. Time until death from each visit was modeled as a continuous outcome with visit-level variables (**Table 1**) as predictors. Data shown are the mean ± SD of the testing *R*^2^ computed over ten repetitions of 10-fold cross validation with calculated missing values (imputed using kNN, see section “Materials and Methods”) embedded within the cross-validation procedure for **(A)** GLM and random forest models at the value of the tuning parameters that minimize the cross-validation RMSE and are plotted over the number of components in the model for several dimensionality reduction techniques (see section “Materials and Methods”). **(B)** The out-of-bag random forest variable importance results for the random forest with all predictors included in terms of the % increase in MSE of prediction upon permutation of the values of each variable. **(C)** Variable importance for a random forest model at the 23rd iteration of the variable clustering algorithm with 14 variable clusters. **(D)** Significance results for variable inclusion in the GLM, as computed by the selective inference method (see section “Materials and Methods”).

Explicitly, time until death from each visit was modeled as a continuous outcome with visit-level variables (**Table 1**) as predictors. Because the visits are treated independently, the resulting predictions are not attached to the known history of a given patient, making them suitable for exploratory population-level insight. The two primary predictive models, which included all 38 metrics shown in **Table 1**, included the GLM illustrated in **Figure 4A** as, “Lasso,” and the full random forest model, “Full-RF.” Additionally, dimensionality reduction techniques were used to construct reduced random forest models.

#### Survival Regression Model Performance

The full random forest regression model, “Full-RF,” for time until death with all 38 visit-level variables (**Table 1**) attains a testing RMSE of 547.73 ± 45.94 days and a testing *R*^2^ of 0.524 ± 0.065 over ten repetitions of 10-fold cross validation, greatly outperforming the GLM, “Lasso,” with a testing RMSE of 697.07 ± 47.10 days and a testing *R*^2^ of 0.216 ± 0.037 (**Figure 4A**) for a mixing parameter α = 0.01 and a regularization parameter λ = 6.629. The out-of-bag random forest variable importance for the full model in terms of the % increase in MSE (**Figure 4B**) indicates that age is the most important of the visit-level variables for the prediction of time until death. Following age, the duration from onset and diagnosis, patient height, % predicted of FVC, and NIF are the next most important variables. None of the muscle scores rank in the top ten in the variable importance list, although the ALSFRS total and ADL sub-score, which moderately correlate with muscle scores, are ranked at 10 and 11, respectively. The patient sex and onset type rank near the bottom of the variable importance list at rank 31 and 37, respectively.

#### Predictive Performance of Dimensionally Reduced Models

Examination of reduced models (models constructed using dimensionality reduction techniques, which utilize fewer variables to predict survival) provide additional insight into the full data structure and relationships among the underlying clinical metrics. The performance of the full random forest model, “Full-RF,” was analyzed under various dimensionality reduction settings to determine if a reduced representation can perform as well as the full model. The random forest models built off of principal component and PLS projections, “PCA-RF” and “PLS-RF,” fail to replicate the performance of the full random forest model (**Figure 4A**). The model built by stepwise inclusion of the variables by variable importance measures, “importance,” performs within one standard deviation of the full model with only four components (age, duration from onset, duration from diagnosis, patient height); applying supervised feature clustering, performance is consistent with the full model through 23 merging steps at which point there are fourteen variable clusters in the model as components, *k* (**Figure 4A**). Variable importance ranking for this reduced 14-component model (**Figure 4C**) compares favorably with the full model (**Figure 4B**).

#### Variable Combination Experiment Identifies Key Clinical Concepts

**FIGURE 5:**
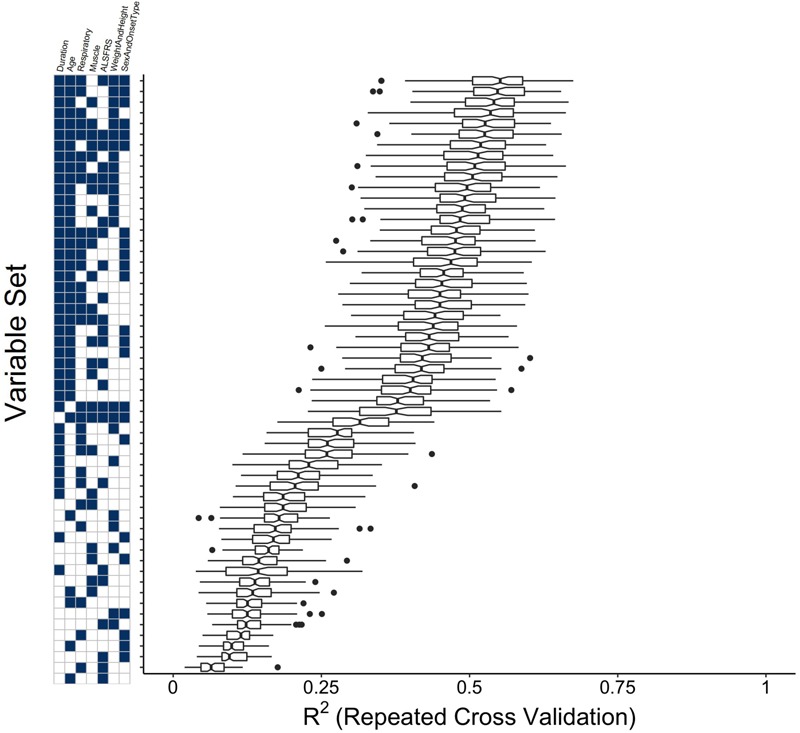
Clinical concept variable grouping combination experiment to compare the performance of reduced models to the full random forest model. Variables were grouped as “Muscle,” “Respiratory,” “Duration,” “ALSFRS,” “Weight and Height,” “Sex and Onset Type” (see section “Materials and Methods”) and all possible combinations of these variable groupings were used as visit-level predictors in random forest models for time until death. Plotted are boxplots of the testing *R*^2^ across the folds of the cross-validation procedure for the model built with the value of m_try_ that minimizes the testing RMSE.

An experiment was performed to determine which concept-based groupings of clinical variables have the most impact in predicting survival: “duration”; “age”; “respiratory,” “muscle” “ALSFRS”; “weight and height”; “onset type and sex.” See Section “Reduced Models Using Dimensionality Reduction” for concept cluster definitions. The evidence that the prediction of time until death is primarily driven by the age and disease duration variables is furthered by the results of the concept-based variable group combination experiment (**Figure 5**). In fact, a model consisting solely of duration and age variables outperforms all models in which only the duration or age is excluded. Beyond that inflection point in the trend, the relative gains in model performance upon inclusion of additional sets are marginal.

#### Model Comparisons Illustrate Importance of Complex Interactions

While the GLM model (“Lasso”) performance results do not compare favorably with the random forest (“Full-RF”) performance results, the *p*-values for variable inclusion (**Figure 4D**) obtained from the selective inference framework (Tibshirani et al., 2014) are statistically interpretable. The results generally support the random forest variable importance, but a few specific departures, such as the insignificance of % predict FVC, likely owed to lasso-prone correlation bias (Tolosi and Lengauer, 2011), and the exaggerated significance of the ALSFRS-R total, collectively reveal the predictive importance of complex interactions and correlation structure accounted by the random forest. Thus, for the purpose of survival prediction, the random forest model is superior.

#### Survival Model Classification Task Performance

**FIGURE 6:**
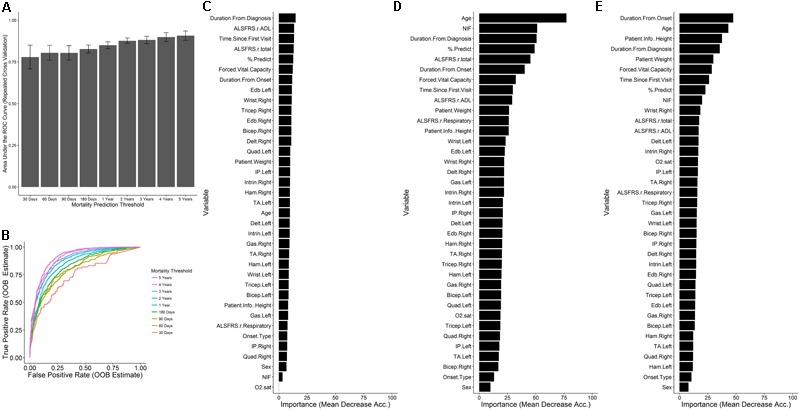
Prediction of mortality as a binary outcome across mortality thresholds. For each visit, the time until death was converted to a binary outcome for thresholds of at 30, 90, and 180 days and 1, 2, 3, 4, and 5 years. For each threshold, random forest classification models were built with ten repetitions of 10-fold cross validation for various levels of m_try_ (see section “Materials and Methods”). **(A)** The mean ± SD of the ROC across the cross-validation procedure for the value of m_try_ with maximal mean cross-validation. **(B)** The out-of-bag prediction for the optimal model for each threshold was used to generate receiver-operating- characteristic (ROC) results across prediction cutoffs. In each case, mortality sooner than the threshold time was considered to be a positive outcome for the purposes of computing performance metrics. The variable importance was assessed for the models at **(C)** 30 days, **(D)** 1 year, and **(E)** 5 years.

The random forest survival model regression task was reframed as multiple classification tasks where the target response is a binary indicator of whether the time of until death for the visit is less than the specified mortality threshold. Mortality thresholds of 30, 60, 90, and 180 days, and 1, 2, 3, 4, and 5 years were considered (**Table 2**). The AUC of the ROC estimate over the cross-validation procedure (**Figure 6A**) tends to increase as a function of the mortality threshold (30 days: 0.77 ± 0.0713; 1 year: 0.85 ± 0.0202; 5 years: 0.907 ± 0.028). The out-of-bag ROC estimate for the final model (**Figure 6B**) demonstrates the tradeoff between the true positive rate (sensitivity) and the false positive rate (1-specificity) for each mortality prediction threshold in general correspondence with the cross-validation ROC results. The analogous out-of-bag precision (positive predictive value) and recall (sensitivity) relationship (Supplementary Figure S2A) indicates severe tradeoffs for detection at 30, 60, and 90 days with roughly linear tradeoffs for thresholds at 1 and 2 years. Additionally, the optimal internal decision cutoff, the fraction of decision tree votes required for positive classification, as measured by the out-of-bag *F*_1_ score (Supplementary Figure S2B), tends to increase as the mortality threshold increases due to the class imbalance at higher mortality thresholds.

The formulation of the model in terms of classification tasks across many time thresholds allows for the assessment of the variable importance for each of those thresholds to obtain a measure of the change in variable importance over the course of disease. At 30 days (**Figure 6C**), the magnitudes of the variable importance measures are diminished with respect to those observed at 1 year (**Figure 6D**) or 5 years (**Figure 6E**) and are relatively uniform in value. At 30 days, the top ranking variables are primarily associated with the time since disease start, the ALSFRS-R total and ADL sub-score, and the FVC and % predicted of FVC. In contrast to other models, the age does not appear to be highly informative for classification at 30 days. At 1 year (**Figure 6D**), the age stands out as the most important variable, followed by the NIF, duration from diagnosis, % predicted of FVC, and the ALSFRS total. At 5 years, the influence of measured clinical variables seem to lose out in importance to time based variables, age, weight, and height. For this case, the FVC (rank 6) is most influential respiratory measure, followed by the % predicted of FVC (rank 8) and NIF (rank 9). In no case do any of the muscle scores seem to rank favorably in terms of variable importance and the onset type, and patient sex consistently rank as the least informative variables.

## Discussion

Our novel assessment of clinical ALS disease complexity using both raw clinical data analysis and interactive survival models identifies dynamic, changing correlations among ALS clinical metrics with disease progression. Knowing how the “best” predictors change with time or ALS disease progression is critical for developing future personalized survival models ready for real-time usage in the clinic or population models for clinical trial development. The relationships identified in the raw data and the predictive models illustrate key prognostic insights that can also lead to the development of better ALS assessment metrics.

Unlike most previous predictive computational models of ALS (Atassi et al., 2014; Küffner et al., 2015; Zach et al., 2015), we directly utilized survival and lifespan-based outcome metrics rather than specifying the ALSFRS-R slope as a measure of ALS progression *a priori*. Using survival as the predicted outcome removes any associated interpretative ambiguity. By utilizing survival as the outcome metric, we were able to evaluate the predictive value of the ardently contested ALSFRS-R alongside other in-clinic metrics, such as MRC muscle scores, clinical respiratory measures, and patient characteristics.

Below we summarize our major findings and their clinical significance. We close by discussing how future advances should focus on the identification of independent metrics of ALS progression that acknowledge its underlying dimensional complexity.

### Time Variables Dominate ALS Survival Prediction

The time variables (patient age, time since onset, time since diagnosis, time since baseline visit, disease duration) are the dominant predictors, particularly for extended survival classification (beyond 1 year). Time variables consistently ranked highly in every model scenario, and the clustered variable experiment (**Figure 5**) showed that they are the single most-important variable group needed to accurately predict survival; that is, time variables provide information that cannot be ascertained from other metrics. As shown in previous work (Fournier and Glass, 2015), time since baseline visit was the most consistent of the time predictors.

### Dominant Clinical Predictors Change With Progression

The best predictors change over time and disease progression (**Figure 6**). For example, functional muscle degeneration (ADL sub-score of the ALSFRS-R, MRC muscle scores) correlates with early disease progression whereas quantifiable respiratory losses (namely % predict FVC but also NIF, O_2_ sat, etc.) correlate better with survival. Height and weight do not factor in to survival classification early on, but are more influential for predicting survival beyond 2 years. Patient age is not important for survival classification at 30 days, but is important at 1 year and beyond. In summary, considering temporal changes in variable importance is key for developing accurate predictions at each disease stage or survival classification. Finally, changes of variable importance with time or disease progression could explain discourse in the field over the importance of specific predictors.

### ALSFRS-R Is Not Necessarily the “Gold Standard”

Revised ALS Functional Rating Scale is typically quite significant in linear models that do not consider complex interactions (**Figure 4D**), which is how its impact has been previously assessed by those citing its veracity (Cedarbaum et al., 1999; Kimura et al., 2006; Kollewe et al., 2008). However, others (Voustianiouk et al., 2008; Franchignoni et al., 2013, 2015; Mandrioli et al., 2015) have acknowledged the concerns of ALSFRS-R validity as a primary outcome metric or as stand-in for survival.

The ALSFRS-R is not highly ranked in any of the random forest models with the exception of the random forest for survival classification at 30 days (**Figure 6C**). Our results reveal that the ALSFRS-total is more discriminatory for patients near death, and its clinical significance is very much tied to the slope of decline in individual patients versus a population-level effect. As such, ALSFRS-R should not be used as the primary or solo outcome metric for population-level models.

Notably, there are complex incongruences in the ALSFRS-total, and especially the ALSFRS-R respiratory sub-score, which is consistently lower ranking or insignificant (**Figures 4, 6** despite clear, consistent significance in the % predict FVC. Our findings corroborate the inability of the ALSFRS-R total score (Franchignoni et al., 2013, 2015; Mandrioli et al., 2015), and even the ALSFRS-R respiratory sub-score (Pinto and de Carvalho, 2015) to represent true progression due to multidimensionality (Franchignoni et al., 2013, 2015) and heterogeneity (Voustianiouk et al., 2008; Mandrioli et al., 2015). Re-evaluation of how sub-scores or ALSFRS-R questions are combined could increase the significance of this universal ALS clinical metric.

### Respiratory, Upper and Lower Muscle Function Losses Are Independent

The raw clinical correlations and the results of all models clearly reveal that upper motor and lower motor losses occur independently, a result that had been theorized (Ravits et al., 2007) but not yet fully embraced by the field. Moreover, the decline in respiratory function occurs predominantly independently from the upper and lower functional muscle degeneration. As noted above, survival prediction is tied more strongly to respiratory metrics, especially % predict FVC, whereas early disease progression is tied more closely to functional muscle degeneration. Respiratory decline has received nearly unanimous support by the field as key survival predictor (Magnus et al., 2002; Fournier and Glass, 2015; Küffner et al., 2015; Mandrioli et al., 2015).

It was of particular interest to assess the predictive value of the MRC muscle scores, as they were not recorded in the PRO-ACT (Atassi et al., 2014) or prior analyses (Kent-Braun et al., 1998; Magnus et al., 2002). Our cumulative findings suggest that no single muscle or group or muscles is independently predictive of survival. However, the independent segregation of the upper and lower muscle scores, as well as the correlation of muscle degeneration to onset, means that individual or aggregate muscle scores could still be valuable for the development of early-stage ALS progression assessments.

Interestingly, a recent meta-analysis of SOD1-G93A ALS mouse data showed that while some treatments, like those targeting oxidative stress, could decrease functional decline by an average of 59.6%, only a 10% average difference in survival was noted; this also suggests that muscle function, while important to quality of life and a key target of ALS disease, is likely not the primary, direct contributor to survival (Bond et al., 2018).

### Onset Type and Gender Are Not Independent Predictors of Survival

Qualitative observations suggest bulbar patients and/or males have shorter disease duration (Magnus et al., 2002; Testa et al., 2004; Mandrioli et al., 2006; Küffner et al., 2015). However, the raw data and all model scenarios reveal that onset type and gender are not independent predictors of survival. Like the DREAM models (Küffner et al., 2015), onset type and gender always rank near the bottom in variable importance. Onset type closely aligns with the information provided by age (shown in **Figure 3**); given bulbar onset ALS patients tend to be older, this finding is intuitive (Coan and Mitchell, 2015). Similarly, females tend to have a longer lifespan than males even in the general [non-ALS] population (Mitchell et al., 2015b); thus, studies citing gender differences (McCombe and Henderson, 2010) are likely tied to either age and/or gender-biased treatment etiology (Pfohl et al., 2015), rather than independent gender effects.

### Height and Weight Have Varying Significance

Height and weight have varying effects, which could explain contested literature (Paganoni et al., 2011; Reich-Slotky et al., 2013; Küffner et al., 2015). Height and weight are, unsurprisingly, intertwined and appear to be especially helpful for extended survival classification, such as at 5 years (**Figure 6E**). In the presented population-level survival prediction models, height appears to be important and thus, could either be a stand-alone population-level predictor (e.g., perhaps due to its co-correlation with motoneuron length) or, in contrast, height could simply a stand-in proxy for weight. Weight, itself, has little predictive value in the illustrated analysis. However, in the present analysis, weight was based upon independent visit records without historical context from a specific patient (e.g., slope of weight decline). Other studies have stated that the slope of weight decline with ALS disease progression is likely more important (Küffner et al., 2015; Hollinger et al., 2016) in terms of predictive power. The impact of weight is complicated by the fact that obesity is tied to earlier ALS onset age (Hollinger et al., 2016).

### Complexity Is the Key to Unlock ALS

A viewpoint change is needed to acknowledge, based on increasing evidence, that ALS is a multi-faceted, inherently complex and system-level disease that has no one single root cause (Coan and Mitchell, 2015; Irvin et al., 2015; Kim et al., 2015; Hollinger et al., 2016). A complex system cannot necessarily be represented by the sum of its individual parts. We illustrated that the ALSFRS-R score, itself, is a low-level example of a metric flawed by the multidimensional asymmetric sum of its parts (Franchignoni et al., 2013, 2015). The identification of a complete set of independent predictors that fully explain ALS “system” variance is paramount. Other variables shown to have quantifiable commonalities among ALS sub-populations, such as antecedent disease (Hollinger et al., 2016), genetic make-up (Hrastelj and Robertson, 2016), blood chemistry (Küffner et al., 2015), physical activity (Gallo et al., 2016), environmental factors (McCombe and Henderson, 2010), and the prognostic value of imaging as identified by recent studies (Baldaranov et al., 2017; Grolez et al., 2018; Menke et al., 2018) could account for remaining variance.

We are still far from developing a mechanistic representation of the complex system of multi-factorial pathophysiological processes (Kim et al., 2015) involved in the progression of ALS, although dynamics and regulatory instability (Irvin et al., 2015; Hollinger et al., 2016) have been hypothesized to be key to treatment prediction (Mitchell and Lee, 2012). A complete disease model is inherently hierarchical (Selinger et al., 2003) and typical observational clinical measures exist as emergent properties at the highest level of that hierarchy with no clear explanatory relationship to the complex underlying pathology. For the practical purposes of personalized clinical prediction of ALS, full specification of all aspects of that complete hierarchical system may not be essential, as recently shown with a personalized prediction model (Westeneng et al., 2018). However, any model built solely off observational clinical measures may be fundamentally deficient in fully explaining the heterogeneity in ALS progression, regardless of modeling strategy. Future work must link lower-level mechanisms to higher-level emergent predictors. Such linking will require identification of common system-level relationships and instabilities (Irvin et al., 2015; Hollinger et al., 2016) that are present in the disease regardless of ALS genotype, phenotype, or initiating mechanism.

## Ethics Statement

This study was carried out in accordance with the recommendations and approval of the Institutional Review Board of Georgia Institute of Technology and Emory University. A waiver for consent was granted for this retrospective study, which utilized de-identified an anonymized data from deceased patients.

## Author Contributions

SP contributed to study design, model construction, statistical analysis, results interpretation, drafted the initial manuscript, and review of content. RK assisted in data collection, analysis, results interpretation, and review of content. GC managed data quality control, assisted in results interpretation, and reviewed the content. CM contributed to project oversight, study conception, study design, data collection protocol, results interpretation, wrote the final manuscript, and review of content.

## Conflict of Interest Statement

The authors declare that the research was conducted in the absence of any commercial or financial relationships that could be construed as a potential conflict of interest.
