# Supplementary material for: Unraveling the Complexity of Amyotrophic Lateral Sclerosis Survival Prediction
Source: Front Neuroinform. 2018 Jun 14;12:36. doi: 10.3389/fninf.2018.00036 (PMC6010549; doi:10.3389/fninf.2018.00036)
Supplement: Supplementary file 1 [file Data_Sheet_1.PDF]

Supplementary Figures

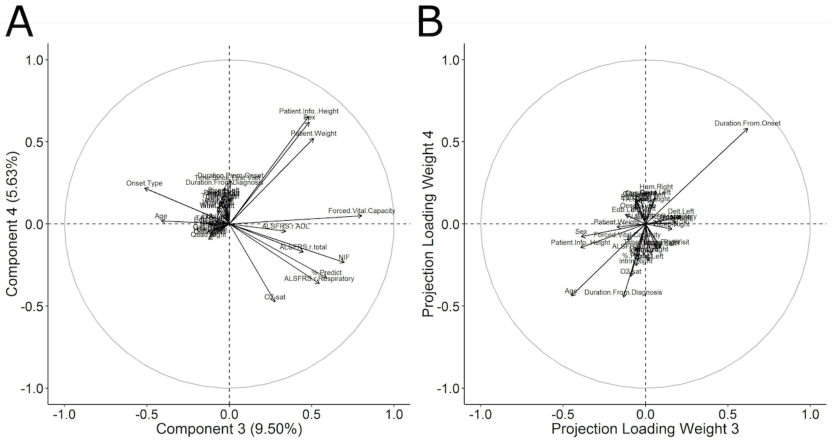

Figure S1. Variable projections onto 3rd and 4th principal components and partial least squares weights. (A) The visit-level predictor dataset was imputed and then decomposed with principal component analysis and the variable projections onto the 3rd and 4th principal component shown. (B) A partial least squares model with the time until death as response was generated and the columns of the weight matrix visualized as variable projections for the third and fourth columns.

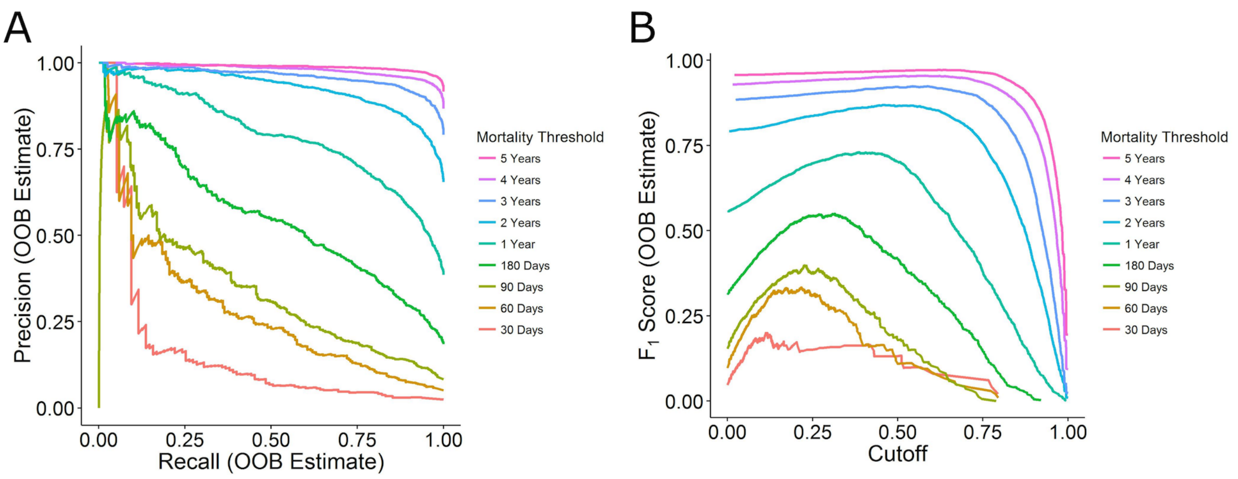

Figure S2. Prediction of mortality as a binary outcome across mortality thresholds for visit records. For each visit, the time until death was converted to a binary outcome for thresholds of at 30, 90, and 180 days and 1, 2, 3, 4, and 5 years. For each threshold, random forest classification models were built with ten repetitions of ten-fold cross validation for various levels of m_{try} (see methods). The out-of-bag prediction for the optimal model for each threshold was used to generate (A) Precision-Recall curves, and (B) F-measure results across prediction cutoffs. In each case, mortality sooner than the threshold time was considered to be a positive outcome for the purposes of computing performance metrics.
